# Short-Term Plasticity at Olfactory Cortex to Granule Cell Synapses Requires Ca_V_2.1 Activation

**DOI:** 10.3389/fncel.2018.00387

**Published:** 2018-10-26

**Authors:** Fu-Wen Zhou, Adam C. Puche, Michael T. Shipley

**Affiliations:** Department of Anatomy and Neurobiology, Program in Neurosciences, University of Maryland School of Medicine, Baltimore, MD, United States

**Keywords:** short-term synaptic plasticity, Ca_V_2.1 channels, feedback projections, olfactory cortex, olfactory bulb, light-gated cation channel channelrhodopsin-2

## Abstract

Output projections of the olfactory bulb (OB) to the olfactory cortex (OCX) and reciprocal feedback projections from OCX provide rapid regulation of OB circuit dynamics and odor processing. Short-term synaptic plasticity (STP), a feature of many synaptic connections in the brain, can modulate the strength of feedback based on preceding network activity. We used light-gated cation channel channelrhodopsin-2 (ChR2) to investigate plasticity of excitatory synaptic currents (EPSCs) evoked at the OCX to granule cell (GC) synapse in the OB. Selective activation of OCX glutamatergic axons/terminals in OB generates strong, frequency-dependent STP in GCs. This plasticity was critically dependent on activation of Ca_V_2.1 channels. As acetylcholine (ACh) modulates Ca_V_2.1 channels in other brain regions and as cholinergic projections from the basal forebrain heavily target the GC layer (GCL) in OB, we investigated whether ACh modulates STP at the OCX→GC synapse. ACh decreases OCX→GC evoked EPSCs, it had no effect on STP. Thus, ACh impact on cortical feedback is independent of Ca_V_2.1-mediated STP. Modulation of OCX feedback to the bulb by modulatory transmitters, such as ACh, or by frequency-dependent STP could regulate the precise balance of excitation and inhibition of GCs. As GCs are a major inhibitory source for OB output neurons, plasticity at the cortical feedback synapse can differentially impact OB output to higher-order networks in situations where ACh inputs are activated or by active sniff sampling of odors.

## Introduction

Sensory processing depends on environmental context and the past experiences of an individual. The neural encoding of these past experiences into “memory” is generally thought to occur via synaptic plasticity. Synaptic plasticity within the olfactory bulb (OB) and olfactory cortex (OCX) is thought to be involved in the sensory processing underlying olfactory memory (Wilson et al., 2004). OB mitral/tufted cells (M/TCs) project to the OCX where they synapse the apical dendrites of principal cells (Carson, 1984; Otazu et al., 2015). Olfactory cortical pyramidal cells, in turn send dense excitatory feedback projections to the main OB (Shipley and Ennis, 1996; Ennis et al., 2015). Plasticity changes at any of these synapses could modulate sensory processing based on past experience.

Granule cells (GCs) are the major synaptic targets of centrifugal fibers from OCX (Davis and Macrides, 1981; Luskin and Price, 1983; Carson, 1984; Shipley and Adamek, 1984; Balu et al., 2007; Boyd et al., 2012). GCs also receive feedback glutamatergic synaptic projections from anterior olfactory nucleus/cortex (Markopoulos et al., 2012). GCs regulate the activity of OB output neurons, M/TCs by way of reciprocal dendrodendritic synapses. M/TCs excite GCs, which in turn, reciprocally inhibit M/TCs (Isaacson and Strowbridge, 1998; Mori et al., 2009). Optical activation of OCX pyramidal cell axons/terminals in OB evokes excitatory postsynaptic currents (EPSCs) in GCs (Boyd et al., 2012). Electrical stimulation of cortical feedback projections to the OB can evoke long- (Cauthron and Stripling, 2014) and short-term (Balu et al., 2007) plasticity at the OCX→GC synapse.

Short term plasticity (STP) is widely believed to play an important role in essential neural functions, including sensory processing (Abbott and Regehr, 2004; Wilson et al., 2004; Li et al., 2018). For most neurons the key determinant of STP is calcium influx through presynaptic P/Q voltage-dependent calcium channels (Ca_V_2.1; Mochida et al., 2008; Catterall et al., 2013). Ca_V_2.1 channels show slow and fast modes of gating, voltage dependent open probability, slow inactivation and voltage dependent steady-state inactivation (Luvisetto et al., 2004). Ca_V_2.1 channels are highly expressed throughout the OB and are especially concentrated in presynaptic terminals (Westenbroek et al., 1995).

Ca_V_2.1 channels can also be targets for modulation by transmitters such as acetylcholine (ACh). Activation of ACh receptors reduces calcium entry into the presynaptic terminal to decrease neurotransmission at basket cell synapses in mouse CA1 hippocampus (Lawrence et al., 2015) and at striatal projection neuron synapses (Perez-Rosello et al., 2005). Cholinergic projections from diagonal band (DB) complex in the basal forebrain (Macrides et al., 1981; Luskin and Price, 1983; Nickell and Shipley, 1988) terminate throughout the OB (Ichikawa and Hirata, 1986; Kasa et al., 1995; Rothermel et al., 2014). Many of these cholinergic axons terminate GC spines. Electrical stimulation of DB *in vivo* and ACh applied in slices regulates GC inhibition of mitral cells (Nickell and Shipley, 1988; Castillo et al., 1999). This could be due to direct cholinergic synapses onto GCs or onto presynaptic terminals of glutamatergic excitatory projections from OCX, or both. ACh could directly modulate Ca_V_2.1 channels at either synaptic site, or act independently of Ca_V_2.1 channels to modulate the OCX→GC synapse. The present research addresses these issues.

We used light-gated cation channel channelrhodopsin-2 (ChR2) to investigate plasticity of EPSCs at the OCX→GC synapse. We report that selective activation of this synapse generates strong, frequency-dependent STP. The plasticity requires activation of Ca_V_2.1 channels on OCX→GC axon terminals. We further report that, although ACh decreases OCX evoked EPSCs, it has no effect on STP.

## Materials and Methods

Male and female transgenic vGLUT2-Cre mice from Jackson Laboratory (120 ± 5 days old, *n* = 22 mice) were used. Animals were maintained with a standard 12 h light/dark cycle and *ad libitum* access to food and water. All experimental procedures were performed in accordance with protocols approved by the University of Maryland Institutional Animal Care and Use Committee.

ChR2 was expressed by injection of Cre-inducible adeno-associated virus serotype 9 (AAV2.9) as described previously (Liu et al., 2013). Briefly, the skull was exposed and four small holes (~0.5 mm diameter; coordination: Bregma, +1.0; lateral, ±2.8; vertical, −4.0 and Bregma, +1.5; lateral, ±2.5; vertical, −3.8; Franklin and Paxinos, 2008) were drilled for injections in OCX. The AAV2.9, carrying fusion genes for ChR2 and enhanced yellow (AAV2.9-hSyn-hChR2(H134R)-EYFP; Liu et al., 2016), were injected at 8–12 weeks postpartum. Injections were performed of 0.25 μl over 5 min per injection site.

To increase the yield for the electrophysiology/pharmacology experiments, virus (total ~1.0 μl) was injected into piriform cortex of both hemispheres. In both hemispheres, infected pyramidal cells appeared to be confined to piriform cortex. However, as we cannot definitively rule out some undetected spread into AON, thus we designate the injection site as “OCX” rather than “piriform cortex.”

After at least 3 weeks for ChR2-EYFP fluorescent protein expression, acute OB slices were cut as previous described (Zhou et al., 2016). Briefly, OB was quickly dissected out and horizontal slices (350 μm) were cut with a VT1200S Vibratome in 4°C oxygenated (95% O_2_–5% CO_2_) cutting solution containing (in mM): 204.5 sucrose, 3 KCl, 1.25 NaH_2_PO_4_, 25 NaHCO_3_, 2.6 MgCl_2_, 10 D-glucose. The slices were then transferred to a holding chamber containing the oxygenated extracellular solution (in mM): 125 NaCl, 2.5 KCl, 1.25 NaH_2_PO_4_, 25 NaHCO_3_, 1.3 CaCl_2_, 1.3 MgSO_4_, 10 D-glucose.

After at least 1 h incubation at 23°C, slices were transferred to recording chamber and perfused at 5 ml/min with extracellular solution. Recordings were made at 30°C under visual guidance of a BX50WI (Olympus) fixed-stage upright microscope equipped with near-infrared differential interference contrast optics. Conventional whole cell patch clamp techniques were used as previously described (Zhou et al., 2009). Records were performed from GCs located in the GC layer (GCL). Patch electrodes had resistances of 7–8 MΩ when filled with an internal solution containing (in mM): 120 CsCH_3_SO_3_, 5 EGTA, 10 HEPES, 5.5 MgCl_2_, 3 Na_2_-ATP, 0.3 Na_3_-GTP, 10 Na-phosphocreatine and 0.1% biocytin (280 mOsm and pH was adjusted to 7.3 with CsOH). MultiClamp 700A amplifiers, pClamp 9.2 software and Digidata 1322A interface (Axon Instruments) were used to acquire and analyze data. Signals were digitized at 5–20 KHz and analyzed offline. D-2-amino-5-phosphonopentanoic acid (D-AP5) and 2,3-dihydroxy-6-nitro-7-sulfamoyl-benzo[f]quinoxaline-2,3-dione (NBQX) were purchased from Tocris and other chemicals were from Sigma.

Fibers from glutamatergic neurons in OCX with ChR2 expression were activated by green laser optical stimulation and the evoked EPSCs recorded at holding potential of −60 mV. Laser light was generated by a 100 mW, 473 nm, diode-pumped, solid-state laser MBL-III-473 (Optoengine) and gated with a high speed laser shutter LST200 (NMLaser Products). Laser beam from fiber tip was calibrated with a PM20A power meter (ThorLabs). Laser beam was delivered from a multimode optical fiber with opening diameter of 25 μm (0.1 NA, ~7° beam divergence). The optical stimulation pulses (2 ms) were triggered by TTL electrical pulses generated by a PG4000A digital stimulator (Cygnus Technology). The laser light output was 0–8.55 mW (or 0–17.4 × 10^−3^ mW/μm^−2^ with the tip of a 25-μm core diameter fiber, 1 mW corresponding to 2 × 10^−3^ mW/μm^−2^). The interstimulus intervals (or stimulation frequencies) varied from 50 ms to 500 ms (or 20–2 Hz) controlled by a MultiClamp 700A commander (Molecular Devices, San Jose, CA, USA).

After electrophysiological recordings, staining was performed as described previously (Zhou and Roper, 2011). Briefly, brain slices with bicytin-filled GCs were fixed in 4% paraformaldehyde (PFA) in 0.1 M phosphate buffer (PBS) at 4°C overnight or longer. Slices were incubated with 4 μg/mL Alexa Fluor 546 streptavidin for 2 h at room temperature. After washing in PBS, slices were incubated with DAPI (300 nM) for 1 h. Slices were then mounted on glass slides, coverslipped with a DABCO based antifade media and imaged. Digital microscopy images were captured using a FluoView500 confocal microscope (Olympus).

Data were analyzed with Clampfit 9.2 (Molecular Devices, San Jose, CA, USA). Peak amplitude was measured as the difference between the baseline current level and the peak of the EPSC. Rise time was defined as the time taken for the current to rise from 10% to 90% of EPSC peak amplitude; decay time was the time to decrease from 90% to 10% EPSC peak amplitude. The paired pulse ratio (PPR) was calculated as the ratio of the peak amplitude of the test (second) EPSC to that of the control (first) EPSC. Statistical analysis and both graphs and plotting were completed with Origin 2018 (Origin Lab). All values were expressed as mean ± SEM. Paired *t*-tests and ANOVA were used to compare data.

## Results

### Frequency Dependent Short-Term Plasticity

**Figure 1:**
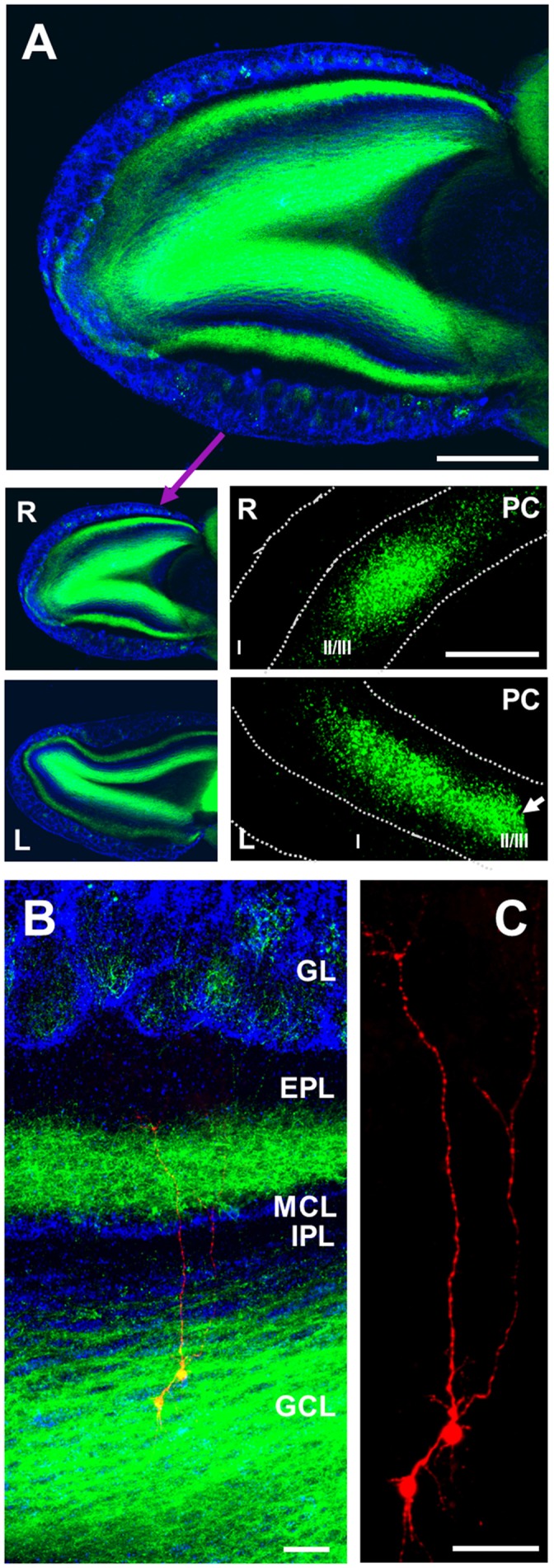
Anterograde labeling of cortico-bulbar glutamatergic axons. **(A)** Staining of channelrhodopsin-2 (ChR2; *green*) and DAPI (*blue*) showing distribution of labeled axons (*green*) in different layers of right (R) olfactory bulb (OB) with the densest labeling in granule cell layer (GCL), and second densest labeling in inner part of external plexiform layer (EPL). Limited fiber labeling is present in glomerular layer (GL), superficial EPL, internal plexiform layer (IPL) and mitral cell layer (MCL). Left (L) OB is also shown (low panel) a similar distribution of labeled axons from olfactory cortex (OCX). Note that left bulb was from cutting level of around 1,000–1,350 μm, and right bulb from around 300–650 μm to dorsal surface of OB. The right and left OCX with infected pyramidal cells are also shown (low panels), but the left one was cut to reduce slice size during the cutting process (white arrow). Scale bar: 400 μm in upper panel, and 1,000 μm in lower panel.** (B)** Triple staining of DAPI (*blue*), ChR2 (*green*) and biocytin (*red*) filling two patch clamped GCs with their somata located in the GCL and apical dendritic trunks projecting to ramify in the EPL. **(C)** Higher magnification showing the biocytin filled cells exhibit the classic dendritic arbor of GCs. Scale bar in **(B,C)**: 50 μm.

To investigate glutamatergic synapses from OCX to GCs in the OB, we expressed ChR2 by injecting the adenoassociated virus AAV2.9-hSyn-hChR2(H134R)-EYFP into OCX of vGLUT2-Cre mice. This drives Cre-dependent co-expression of the light-activated channel ChR2 and enhanced yellow fluorescent protein (YFP). YFP-labeled glutamatergic cells were observed in layers II-III of OCX as expected for expression of vGLUT2 (Figure 1A). Labeled axons/terminals were predominantly in the GCL and inner part of external plexiform layer (EPL), with only sparse fibers present in the glomerular layer (GL; Figures 1A,B) consistent with previous reports of olfactory cortical projections (Luskin and Price, 1983; Shipley and Adamek, 1984; Diodato et al., 2016; Mazo et al., 2016). Interestingly, labeled fibers were present in the inner parts of the EPL in the majority of our cases, this layer has been considered a sparse target for OCX innervation. OCX pyramidal cells do project with differential density to different layers of the main OB, and the viral injection targets/vGlut expressing cells may have different distribution patterns than those of earlier tract tracing studies. Moreover, the number of OCX cells expressing ChR2 may have been greater than previous studies. The EPL labeling could also be due to undetected spread into AON (see “Materials and Methods” section).

**Figure 2:**
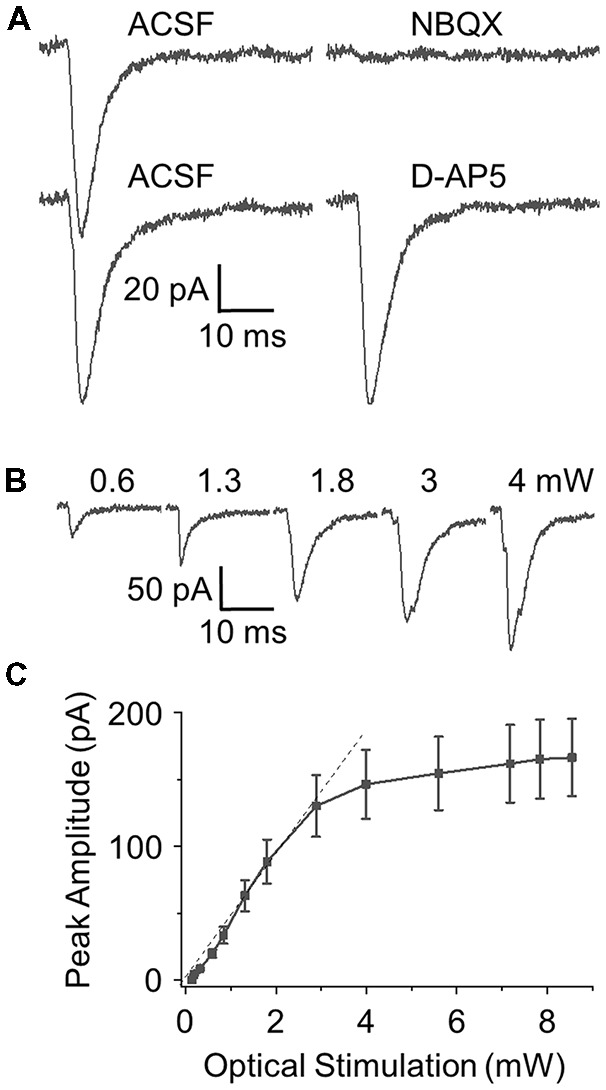
Optical stimulation of ChR2 expressing OCX axons elicits robust excitatory synaptic currents (EPSCs) in GCs. **(A)** Optically evoked GC EPSCs are blocked by the AMPA/kainate receptor antagonist 2,3-dihydroxy-6-nitro-7-sulfamoyl-benzo[f]quinoxaline-2,3-dione (NBQX; 10 μM, *n* = 5). No change in EPSC was observe with block of NMDA receptors with the antagonist D-2-amino-5-phosphonopentanoic acid (D-AP5; 50 μM, *n* = 5). The rise time, decay time and peak amplitude was 1.77 ± 0.27 ms, 10.33 ± 1.34 ms and 104.31 ± 14.33 pA in ACSF and 1.71 ± 0.32 ms, 10.02 ± 1.29 ms and 109.8 ± 13.76 pA in D-AP5, respectively, and they were no difference. **(B)** Representative EPSCs elicited by varying optical power from 0 mW to 4 mW. **(C)** Curve fitting of the power-response profile shows a linear response from 0 mW to 3 mW with response amplitude plateauing out to 8 mW (*n* = 5).

We recorded cells located within the GCL, filling all cells with biocytin followed by *post hoc* immunohistochemical staining to verify that they matched classic GC morphology (Figure 1C). GCs were held at −60 mV and ChR2-expressing axons and terminals from OCX optically stimulated. In all cells tested light activation of ChR2 fibers evoked a robust EPSC which was completely blocked by AMPA/kainate receptor antagonist NBQX (*n* = 5) but not affected by NMDA receptor antagonist D-AP5 (*n* = 5; Figure 2A). Light intensity was systematically varied to generate an EPSC amplitude–stimulation intensity relationship with EPSC peak amplitude increasing linearly with increments of stimulation intensity from 0 mW to 3.0 mW (Figures 2B,C, *n* = 5). In order to observe either increased or decreased EPSC amplitude in the paired-pulse experiments below, an intermediate stimulation intensity of ~1.7 mW was used.

**Figure 3:**
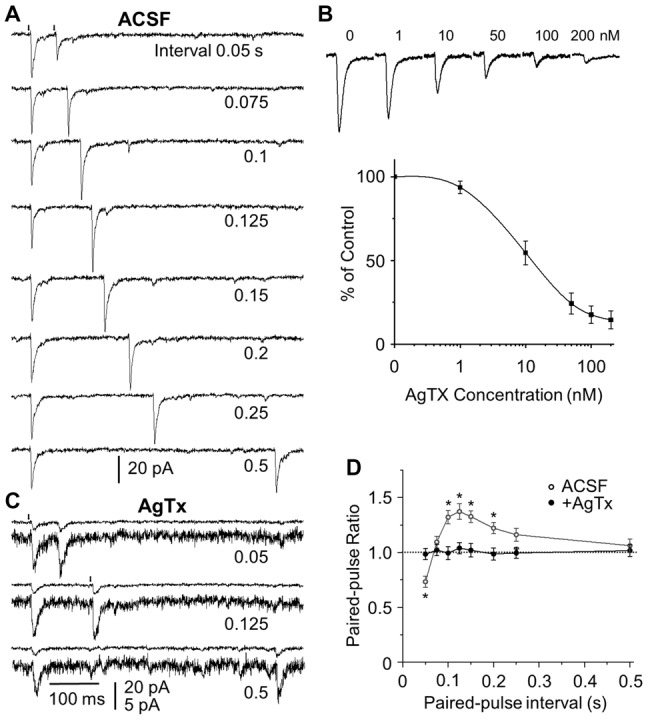
Frequency dependent short-term plasticity (STP) at the OCX to GC synapse requires Ca_V_2.1 calcium channels. **(A)** Representative traces of paired pulse optical stimulation (*arrows*) at interstimulus intervals of 0.05–0.5 s (corresponding to stimulation frequencies of 2–20 Hz) in ASCF. **(B)** Dose response curve for ω-agatoxin IVA. EPSC traces in the presence of bath applied ω-agatoxin IVA (AgTx), the Ca_V_2.1 irreversible antagonist from 0 nM to 200 nM (upper panel) and population data of EPSC peak amplitude expressed as the percentage of the control (0 nM) amplitude (*n* = 4–5). **(C)** Bath application of AgTx (100 nM) reduces EPSC amplitude and eliminates short term synaptic plasticity. To show the difference of the control and test EPSC peak amplitude, three sets of double traces are shown: one upper trace has the same scale as **(A)** and the other one has expanded scale. **(D)** Population data (*n* = 6) showing the paired pulse ratio (PPR) in ASCF (open circle) and in the presence of ω-agatoxin IVA (closed circles). The control EPSCs had the similar peak amplitudes from different stimulation intervals. However, the test EPSC peak amplitude might be different from that of the controls, as shown PPRs different from 1. The rise and the decay time were the same. For example, at the interstimulus interval of 0.125 s/8 Hz, control and test rise time, decay time and peak amplitude was 1.75 ± 0.30 and 1.71 ± 0.27 ms, 9.82 ± 1.57 and 9.73 ± 1.49 ms, 101.42 ± 15.31 and 139.71 ± 17.9 pA in ACSF, and 1.84 ± 0.41 and 1.87 ± 0.39 ms, 9.04 ± 1.72 and 9.11 ± 1.69 ms, 18.34 ± 2.58 and 18.95 ± 2.67 pA in AgTX. Significant difference at *P < 0.01.

Paired pulse optical activation was applied at 2–20 Hz (interstimulus intervals 50–500 ms) and PPRs were calculated. At the highest frequency tested (20 Hz/50 ms), there was a significant short-term depression (ratio 0.733 ± 0.051). As frequency decreased the paired pulse depression (PPD) converted to paired pulse facilitation (PPF). At 13.3 Hz the PPR was close to 1 (ratio 1.095 ± 0.053). However, as frequency decreased into sniff/respiratory ranges facilitation was apparent at 10 Hz (ratio 1.321 ± 0.061), peaking at 8 Hz (ratio 1.373 ± 0.072) before declining back to neutral (ratio 1.062 ± 0.061) at 2 Hz (Figures 3A,D, *n* = 6 for all tested frequencies). This suggests that at paired-pulse frequencies corresponding to sniffing rates (4–10 Hz) there is pronounced short term facilitation of OCX glutamatergic synaptic inputs to GCs, while at resting respiration rates around 2 Hz there is neither depression or facilitation.

### Short-Term Plasticity Requires Ca_V_2.1 Channel Activation

Calcium channels are major contributors to short-term synaptic plasticity (STP) of neurotransmission (Catterall et al., 2013) and Ca_V_2.1 type channels are highly expressed in presynaptic terminals in the OB (Westenbroek et al., 1995). To investigate the role of Ca_V_2.1 channels in STP at the OCX→GC synapse, we examined the effect of the selective, irreversible Ca_V_2.1 channel blocker, ω-agatoxin IVA (Mintz et al., 1992) on PPD/facilitation. Previous slice studies used concentrations ranging from 50 nM to 500 nM in OB (Wang et al., 1996) and other brain regions (Mochida et al., 2008; Nimmrich and Gross, 2012). To determine an effective dose range for the OCX→GC synapse we generated a dose-response curve from 1 nM to 200 nM for reduction of EPSC peak amplitude (*n* = 4–5; Figure 3B). At a low dose of 1 nM there was an amplitude reduction of 6.3 ± 3.8% compared to control while at the high dose of 200 nM EPSC amplitude was reduced by 85.5 ± 5.4%. The half-amplitude reduction (IC50) was at 11.8 ± 1.4 nM. The effect of ω-agatoxin IVA was unchanged upon 30 min washout consistent with irreversible binding.

To test Ca_V_2.1 channel involvement in OCX→GC STP, 50 nM or 100 nM of ω-agatoxin IVA were selected from our dose- response curve to be “strong” but not completely blocking Ca_V_2.1 channels. This compared favorably with doses used to investigate STP in other brain regions (Mochida et al., 2008; Chamberland et al., 2017). Both 50 nM and 100 nM completely blocked STP resulting in a PPR ~1.0 across all frequencies tested from 2 Hz to 20 Hz (*n* = 6; Figures 3C,D, 5B,D). Thus, blocking Ca_V_2.1 channels completely abolishes STP at the OCX→GC synapse indicating that the Ca_V_2.1 channels are necessary for frequency-dependent STP at the glutamatergic OCX→GC synapse.

**Figure 4:**
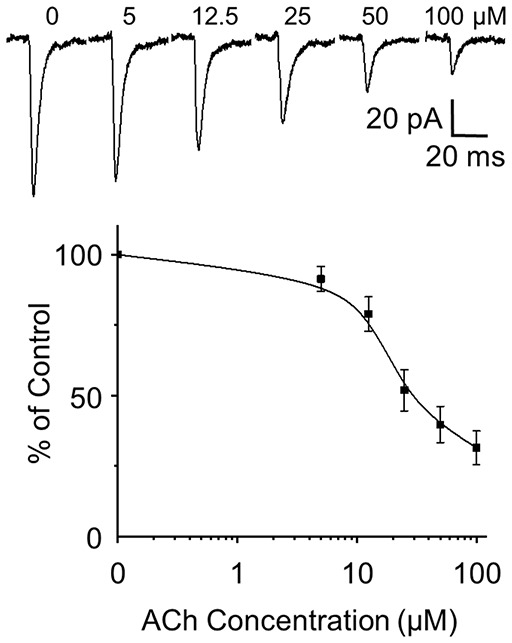
Dose response curve for acetylcholine (ACh). EPSC traces in the presence of bath applied ACh from 0 μM to 100 μM (upper panel) and population data of EPSC peak amplitude expressed as the percentage of the control (0 μM) amplitude (*n* = 4–6).

**Figure 5:**
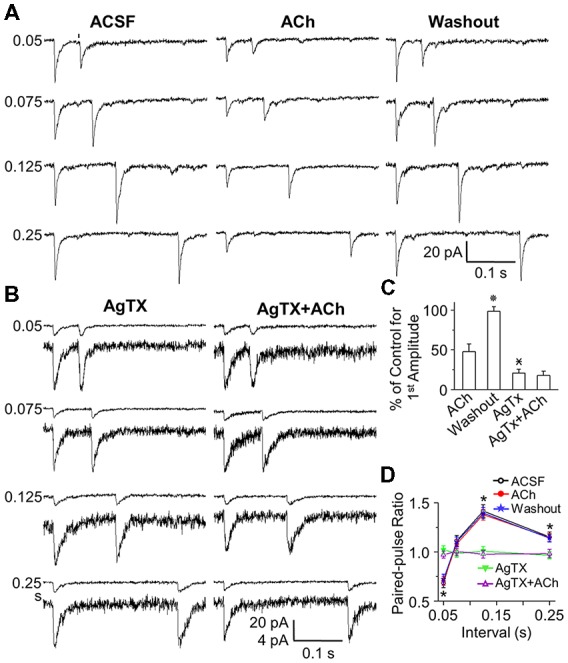
ACh reduces optical stimulation-evoked EPSC peak amplitude, but does not change STP. **(A)** Representative traces of paired pulse optical stimulation (*arrows*) evoked EPSCs at interstimulus intervals of 0.05, 0.075, 0.125 and 0.25 s (corresponding to stimulation frequencies of 20, 13.3, 8 and 4 Hz in ACSF (control), during bath application of ACh (50 μM), and after washout. **(B)** Representative traces of paired pulse optical stimulation evoked EPSCs in the presence of AgTX (50 nM) and AgTx (50 nM) plus ACh (50 μM). **(C)** Population data showing EPSC amplitude as a percentage of ACSF amplitude. **(D)** Population data (*n* = 6) showing PPR under control (open circle), in the presence of ACh (red circle), following ACh washout (star) and in the presence of AgTX (triangle) and AgTX/ACh (open triangle). The control EPSCs were similar, but they might be different from that of the test EPSC peak amplitude. The rise and the decay time were the same. For instance, at interval of 0.125 s/8 Hz, control and test rise time, decay time and peak amplitude was 1.72 ± 0.27 and 1.67 ± 0.33 ms, 10.22 ± 1.47 and 10.07 ± 1.58 ms, 96.84 ± 13.95 and 136.78 ± 16.23 pA in ACSF, and 1.61 ± 0.38 and 1.65 ± 0.43 ms, 9.36 ± 1.66 and 9.43 ± 1.72 ms, 45.42 ± 5.34 and 63.77 ± 5.94 pA in ACh. Statistical significance 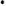
*P* < 0.01 vs. ACh; 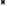
*P* < 0.01 vs. Washout; *P < 0.01 vs. AgTx.

### Acetylcholine Reduces EPSC Amplitude, but Does Not Change Short Term Plasticity

Cholinergic axons from the basal forebrain innervate all the layers of the OB. Recently they have been reported to form synaptic contacts with the dendrites of GCs (Liberia et al., 2015). ACh has also been implicated in modulation of Ca_V_2.1 calcium channels in other brain regions (Perez-Rosello et al., 2005; Lawrence et al., 2015). To investigate a potential role of ACh at the OCX→GC synapse we first compared the dose-response curves to optically evoked OCX→GC EPSCs before and after bath applied ACh. Increasing doses of ACh from 5 μM to 100 μM reduced the amplitude of optically evoked EPSCs with IC_50_ at approximate 28.9 ± 4.3 μM of ACh (*n* = 4–6; Figure 4). We tested 125 μM ACh for three cells showing a similar EPSC peak amplitude (not shown) to 100 μM ACh and treated 100 μM as a presumed saturation concentration for EPSC inhibition.

As ACh and antagonism of Ca_V_2.1 both reduce glutamatergic synaptic transmission from the OCX, we hypothesized that ACh may act on Ca_V_2.1 channels in OCX synaptic terminals. To explore this possibility, we examined whether ACh attenuates STP at the OCX→GC synapse. Bath application of 50 μM ACh decreased the peak amplitude of both the control and test EPSC in a paired pulse experiment and washout of ACh restored EPSC amplitude (*n* = 6; Figure 5A). However, ACh decreased the amplitude of control and test EPSC in a proportional relationship, such that STP was unaltered (Figures 5A,C,D). As in control experiments, high frequency (20 Hz) resulted in a PPD, while slower frequencies (4 and 8 Hz) elicited facilitation (Figures 5A,C). Even ACh at higher concentration (100 μM; *n* = 3 cells) strongly suppressed EPSC amplitude, but did not change PPR (not shown). This suggests that ACh does not act on Ca_V_2.1 channels. To further test this, we applied both ω-agatoxin IVA (50 nM) and ACh (50 μM). Blocking Ca_V_2.1 reduced EPSC amplitude and abolished STP as above. However, addition of ACh did not significantly increase the EPSC attenuation observed with Ca_V_2.1 blocker alone and did not further change PPR (*n* = 6; Figures 5B–D). Taken together, these findings show that ACh is not additive to Ca_V_2.1 channel block, suggesting that ACh’s suppression of the OCX→GC EPSC is independent and upstream of Ca_V_2.1 channels, although we cannot exclude ACh also having some post-synaptic action.

## Discussion

STP is a feature of many synaptic connections in the brain and is thought to be involved in sensory processing and memory formation. The present findings indicate there is strong frequency-dependent, STP at glutamatergic synapses from OCX to OB GCs. We further show that this plasticity requires Ca_V_2.1 channels and is not modulated by centrifugal cholinergic afferents. The OB→OCX→OB feedback loop is a rapid tri-synaptic circuit. The function of this feedback loop is poorly understood but is thought to be fundamental to odor processing and the oscillatory dynamics of OB during exploratory sniffing (Kiselycznyk et al., 2006; Labarrera et al., 2013; Nunez-Parra et al., 2013). The present findings show that cortical feedback part of the circuit exhibits strong short-term facilitation at frequencies corresponding to investigatory sniffing. This could *increase* excitation of GCs and thereby *decrease* the excitability of OB output neurons during bouts of sniffing odorants.

### Short Term Synaptic Plasticity

Electrical stimulation of centrifugal axons projecting into the bulb induces long-term (Gao and Strowbridge, 2009; Cauthron and Stripling, 2014) and STP (Balu et al., 2007) of GC synaptic responses. The origin of the stimulated fibers in these studies is unclear as electrical stimulation activates all input fibers, which comprise a mix of glutamatergic, cholinergic, GABAergic, noradrenergic and serotonergic inputs (Fallon and Moore, 1978; Macrides et al., 1981; McLean et al., 1989; Ennis et al., 2015). The present study used gene-targeted viruses to drive ChR2 expression selectively in glutamatergic OCX→OB axons. We show that there is robust, STP at the OCX→GC synapse. PPD at high frequency (20 Hz, 50 ms inter-stimulus interval) is consistent with models of vesicular availability limiting transmitter release from a second stimulus at a short interval (Wesseling and Lo, 2002; Zucker and Regehr, 2002). The number of synaptic vesicles released depends on the size of readily-releasable pool and the initial probability of release (Fioravante and Regehr, 2011). During high frequency stimulation, the readily releasable vesicle pool is depleted more quickly than it can be replenished. As the number of vesicles in the readily releasable pool becomes rate limiting, subsequent stimuli release fewer vesicles resulting short-term PPD (Zucker and Regehr, 2002).

At resting respiration rates of ≤2 Hz there was neither PPD or PPF, suggesting a “neutral” plasticity setpoint corresponding to quiet breathing. In contrast, at frequencies spanning investigative sniffing rates (4–10 Hz; Wachowiak, 2011) there was robust PPF at the OCX→GC synapse. Thus, whether this synapse shows depression or facilitation is dependent on input frequency. Balu et al. (2007) reported facilitation across all inter-stimulus intervals from 5 Hz to 50 Hz using electrical stimulation, which would activate a mix of transmitter-specific centrifugal fibers from different brain regions. The present experiments used selective optical activation of ChR2 in vGLUT2cre expressing OCX axons/terminals in the OB. The difference in facilitation frequencies in the two studies raises the interesting possibility that there is heterogeneity in STP from different brain regions. Conceivably, axons from posterior piriform cortex or entorhinal cortex may exhibit different frequency properties to STP from the more anterior OCX region in this study. Synchronous activation of all projections could cause net facilitation masking the frequency dependent depression from OCX, whereas selective activation glutamatergic inputs only from OCX in this study exhibits frequency-dependent depression or facilitation.

Mechanisms of STP usually involve presynaptic, voltage-gated calcium channels (VGCCs), including N-type, P/Q type and R-type (Reid et al., 2003; Catterall and Few, 2008; Catterall et al., 2013). The types of VGCCs that control transmitter releases vary with different neuron types, brain regions and species. Ca_V_2.1 channels have been implicated as a major source of Ca^2+^ entry and a key determinant of synaptic release. Because of the steep dependence of transmitter release on calcium, even small activity-dependent decreases or increases in calcium entry by modulation of Ca_V_2.1 channels can lead to significant short-term depression or facilitation (Forsythe et al., 1998; Xu and Wu, 2005; Catterall et al., 2013). Presynaptic Ca_V_2.1 channels cause frequency-dependent, bidirectional synaptic plasticity in superior cervical ganglion neurons: PPD at short stimulation intervals (<50 ms) and facilitation at longer intervals (60–150 ms; Mochida et al., 2008). The present results showed a highly similar frequency-dependent plasticity with depression at short intervals and facilitation at longer intervals. Moreover, selective pharmacologic blockade of the Ca_V_2.1 channels abolished all synaptic plasticity at the glutamatergic OCX→GC synapse, indicating strong dependence on the Ca_V_2.1 channels at this synapse. However, other Cav channels may also be involved in STP and remain to be explored at the OCX to GC synapse.

### ACh Modulation of Cortical Glutamatergic Input

Ach modulation of bulb circuitry is complex with direct and indirect actions upon many of the neuron types in the OB. Activation of muscarinic receptors has been reported to inhibit GC firing, but increase activity-independent transmitter release of GABA from GCs to mitral cells (Castillo et al., 1999). On the other hand, muscarinic receptor activation increases GC excitation by blocking afterhyperpolarization (Pressler et al., 2007). Activation of nicotinic receptors excites both output neurons and multiple classes interneurons (Castillo et al., 1999), including the recently identified deep short axon cells (Burton et al., 2017). Little is known about cholinergic presynaptic modulation of centrifugal axons/terminals in OB. In other brain regions, ACh directly modulates Ca_V_2.1 calcium channels in presynaptic terminals (Perez-Rosello et al., 2005; Lawrence et al., 2015). In the present study ACh in a dose-dependently attenuated OCX terminal-evoked EPSCs in GCs, but did not modulate PPD or PPF. Thus, ACh’s actions appear to be independent of the Ca_V_2.1 channel, although its reduction of EPSC peak amplitude at this synapse suggests it modulates other voltage-gated channels or other components of synaptic machinery. One potential mechanism would be reduction in vesicle content or availability, which would reduce amplitude but not alter the release machinery underlying STP.

Neuropharmacologic-behavior studies suggest that ACh modulates olfactory memory. Blockade of muscarinic receptors impairs short-term olfactory memory (Ravel et al., 2003), while blockade of nicotinic transmission depresses discrimination between similar odorants (Mandairon et al., 2006). Although these studies suggest that cholinergic receptors are involved in STP, they do not identify the neuron type(s) or circuits that mediate complex behavioral processes at the circuit level. Our findings suggest that one of many possible targets is the OCX→GC synapse.

### Function Implications

GCs are activated by dendrodendritic excitation M/TCs, which also send excitatory synapses to OCX neurons. Many OCX neurons project back to the OB and form glutamatergic, excitatory synapses onto GCs. GCs also make reciprocal dendrodendritic synapses with M/TCs and can inhibit M/TCs. Thus, GCs are situated to respond dynamically to multiple sources of synaptic input to shape OB output to other brain regions. Modulation of the OCX→GC synapse could strongly regulate their inhibitory action on M/TCs. Frequency-dependent STP of the OCX→GC synapse as shown may determine the precise balance of excitation and inhibition of GCs. Animals adjust their sniffing according to the salience of odorant stimuli. At quiescent respiration rates (≤2 Hz) there is neither facilitation or depression, but as sniff rate increases the OCX→GC synapse would be progressively facilitated increasing inhibition of OB output neurons. Frequency-dependent enhancement of the OCX→GC synapse might preferentially reduce output neuron firing during investigative sniffing when animals attend to salient stimuli, however it is unclear how prolonged sniffing at high frequency may affect potentiation/depression as synapses may deplete during train stimulation. This could enhance OB signal-to-noise ratios to facilitate odor processing.

## Author Contributions

MS, AP and F-WZ designed the experiments and wrote the article. F-WZ performed and analyzed the experiments.

## Conflict of Interest Statement

The authors declare that the research was conducted in the absence of any commercial or financial relationships that could be construed as a potential conflict of interest.
